# Production, Characterization, and Epitope Mapping of Monoclonal Antibodies Against Different Subtypes of Rabbit Hemorrhagic Disease Virus (RHDV)

**DOI:** 10.1038/srep20857

**Published:** 2016-02-16

**Authors:** Desheng Kong, Jiasen Liu, Qian Jiang, Zuo Yu, Xiaoliang Hu, Dongchun Guo, Qianqian Huang, Meihui Jiao, Liandong Qu

**Affiliations:** 1 Zoonosis of Natural Foci, State Key Laboratory of Veterinary Biotechnology, Harbin Vet Res Institute of Chinese Academy of Agricultural Sciences, Harbin 150001, PR China

## Abstract

In 2010, a new rabbit hemorrhagic disease virus (RHDV) variant, designated RHDV2, was identified for the first time in Italy. Studies have shown that RHDV2 differs from RHDV1 (traditional RHDV) in terms of its antigenic profile and genetic characteristics. The VP60 protein of RHDV is a structural protein that plays important roles in viral replication, assembly, and immunogenicity. In this study, we immunized BALB/c mice with recombinant VP60 proteins from different RHDV subtypes. After three rounds of subcloning, type-specific positive hybridoma clones of RHDV1 and RHDV2 were further identified by an enzyme-linked immunosorbent assay, Western blotting, and an indirect immunofluorescence assay. Finally, three monoclonal antibodies (MAbs) (1D6, 1H2, and 3F2) that only recognize RHDV1, and four MAbs (1G2, 2C1, 3B7, and 5D6) that only recognize RHDV2 were identified. The epitopes recognized by these MAbs were mapped by Western blotting. Sequence analysis showed that the epitope sequences recognized by 1D6, 1H2, and 3F2 are highly conserved (98%) among RHDV1 strains, whereas the epitope sequences recognized by 1G2, 2C1, 3B7, and 5D6 are 100% conserved among RHDV2 strains. The high conservation of the epitope sequence showed that the screened MAbs were type-specific, and that they could distinguish different RHDV subtypes.

Rabbit hemorrhagic disease virus (RHDV) is a member of the family *Caliciviridae*, genus *Lagovirus*, and it causes rabbit hemorrhagic disease (RHD), a disease characterized by high morbidity and high mortality in both wild and domestic adult rabbits^1^. Since first being reported in China in 1984^2^, RHD spread worldwide within a few years^3^. Infected adult rabbits succumb from 48 to 72 h post-infection, and death is often associated with hepatic, intestinal, and lymphoid necroses, as well as massive terminal intravascular coagulation^4^. Like other caliciviruses, RHDV forms 28–32 nm diameter, non-enveloped, icosahedral virus particles that harbor a 7.4-kb positive-sense single-stranded RNA genome that encodes a 257 kDa polyprotein^5^,^6^. The genome of RHDV has two open reading frames (ORFs): ORF1 encodes a polyprotein that is cleaved into non-structural components and the major structural protein, the capsid protein VP60^7^, which is the main target of the host immune defense against RHDV and plays an important role in virus diagnosis and vaccine design^8^; and ORF2 encodes the minor structural protein VP10^9^. VP60 is composed of three domains, including the amino-terminal arm, the shell (S), and the protrusion (P) domains, the latter of which is further divided into the P1 and P2 sub-domains^10^. According to its amino acid sequence, VP60 can be divided into six distinct regions (A–F). The C and E regions are located in the exposed P2 sub-domain and show the highest degree of genetic variation^11^, and they may contain an important epitope for anti-RHDV antibody production. The E region also contains the main antigenic determinants^12^.

More recently, atypical RHD outbreaks in vaccinated rabbits, which have resulted in high mortality rates in young rabbits, appear to be changing the epidemiology of this disease, and they have coincided with the emergence and spread of a new RHDV variant^13^,^14^,^15^. Various terms, such as “new variant”, “RHDVb” and “RHDV2”, have been used to describe the new RHDV variant, as a definitive RHDV nomenclature has not yet been agreed upon^16^. This multiple naming of subclusters has caused confusion in the field, and for this study, we used the nomenclature of RHDV1 (traditional RHDV) and RHDV2 to define different subtypes of RHDV. The average nucleotide identity between RHDV2 and RHDV1-RHDVa is 82.4%, whereas the average amino acid similarity is about 89.2%^17^. Studies have shown that RHDV2 differs from RHDV1 in terms of its antigenic profile and genetic characteristics, although partial cross-protection exists between RHDV1 and RHDV2^17^, highlighting the need of using RHDV2-specific diagnostic assays to monitor the spread of this new virus^18^. It has been reported that several MAbs against RHDV capsid protein fail to react with cognate RHDV2 virions^17^,^19^. Based on a phylogenetic analysis of the *VP60* gene, RHDV2 forms a separate branch, which may indicate that RHDV2 is a new number of the *Lagovirus* genus^17^. To date, RHDV2 has been detected in Spain, France, Great Britain, and Italy^15^,^20^,^21^,^22^. At present, RHDV2 has not been reported in China. Therefore, it is very urgent to study the etiology, epidemiology, diagnosis, and control of RHDV.

The hybridoma technique has the potential to provide an inexhaustible supply of quality monospecific monoclonal antibodies (MAbs)^23^. MAbs can serve as useful diagnostic tools and act as probes for cellular and macromolecular investigations^24^. In the present study, the VP60 proteins of RHDV1 and RHDV2 were used as immunogens to prepare RHDV type-specific MAbs by hybridoma fusion. Finally, three MAbs that specifically recognized RHDV1 and four MAbs that specifically recognized RHDV2 were identified. The epitopes recognized by these type-specific MAbs were also precisely mapped. Type-specific MAb preparation provides the foundation for the establishment of RHDV subtype-specific detection methods, and it has important significance for RHDV epidemiological investigations and phylogenetic analyses.

## Results

### Expression of recombinant VP60 proteins

The entire *VP60* genes of different RHDV subtypes were expressed as histidine-tagged (His-tagged) fusion proteins in *Escherichia coli* BL21 Star(DE3)pLysS. The VP60 proteins were successfully expressed and examined by sodium dodecyl sulfate-polyacrylamide gel electrophoresis (SDS-PAGE) analysis of cell lysates after induction with isopropyl β-D-1-thiogalactopyranoside (IPTG). The VP60 proteins of RHDV1 and RHDV2, pVP60-1 and pVP60-2, respectively, were successfully purified by excising the relevant gel slices and extracting the proteins. Western blotting analysis showed that the recombinant His-tagged VP60 proteins reacted with rabbit hyperimmune serum (for details, see Supplemental Information Figure S1), suggesting that the recombinant VP60 proteins expressed in *E. coli* had good antigenicity and can be used immunogens.

The RHDV1 and RHDV2 VP60 proteins were also expressed via a eukaryotic expression system, and they are referred to as sVP60-1 and sVP60-2, respectively. sVP60-1 and sVP60-2 were expressed using the Bac-to-Bac Baculovirus Expression System after recombinant RHDV1 VP60 baculovirus and recombinant RHDV2 VP60 baculovirus were seeded separately onto *Spodoptera frugiperda* 9 (Sf9) cells. The *VP60* genes of RHDV1 and RHDV2 were also cloned into the eukaryotic expression vector pcDNA 3.1(+) to obtain pcDNA-R1-VP60 and pcDNA-R2-VP60, respectively, which were used to separately transfect HeLa cells. The VP60 proteins of RHDV1 and RHDV2 that were expressed in HeLa cells were named eVP60-1 and eVP60-2, respectively. VP60 expression in Sf9 and HeLa cells was identified by an immunofluorescence assay (IFA). Green fluorescence was detected in Sf9 and HeLa cells after infection or transfection, respectively (for details, see Supplemental Information Figure S2). The results of the IFA suggested that the RHDV1 and RHDV2 VP60 proteins were correctly expressed in Sf9 and HeLa cells.

### Production of MAbs against recombinant VP60 proteins

Female BALB/c mice were separately immunized with purified VP60 proteins from different RHDV subtypes. Hybridomas were obtained by fusing SP2/0 cells with spleen cells from immunized mice. Hybridomas were cultured in 96-well culture plates and subcloned by limiting dilution. Supernatants were assayed 10 days after cell fusion, and wells with confluent hybridomas were initially screened by an indirect enzyme-linked immunosorbent assay (ELISA) for the secretion of antibodies against RHDV. Finally, 20 strains of hybridoma cells against RHDV1 and 15 strains of hybridoma cells against RHDV2 were filtered out after three rounds of subcloning.

### Identification and characterization of RHDV type-specific MAbs

**Figure 1:**
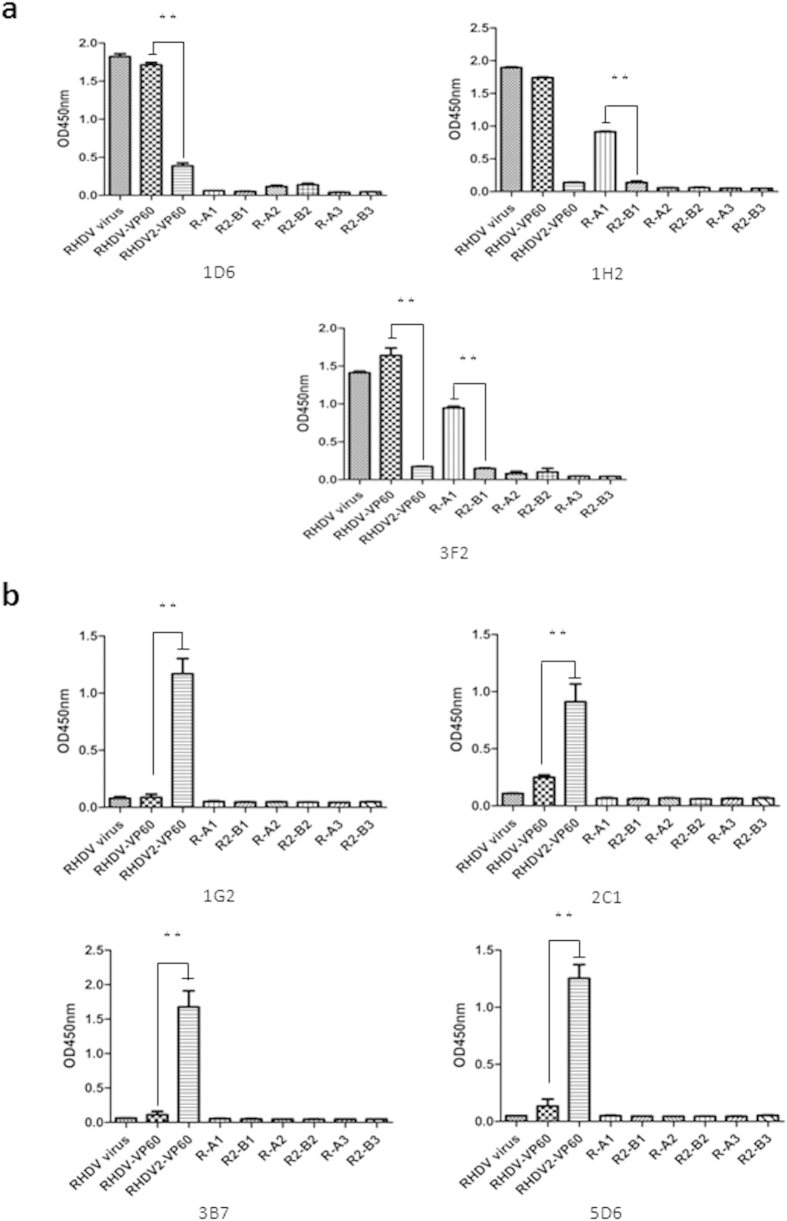
Identification of RHDV type-specific MAbs by indirect ELISA. The RHDV TP strain, pVP60-1, pVP60-2, and six short peptides were used as the detection antigens with positive hybridomas. Among the 20 MAbs against RHDV1, three MAbs, 1D6, 1H2, and 3F2 (**a**), which only recognized RHDV1, were detected. Among the 15 MAbs against RHDV2, four MAbs, 1G2, 2C1, 3B7, and 5D6 (**b**), which only recognized RHDV2, were detected. MAbs 1H2 and 3F2 could also react with R-A1 (amino acids 298–319).

Twenty strains of hybridoma cells against RHDV1 and 15 strains of hybridoma cells against RHDV2 were first identified by indirect ELISA to determine whether the screened MAbs were type-specific. The RHDV TP strain, pVP60-1, pVP60-2, and six short peptides (R-A1, R-A2, R-A3, R2-B1, R2-B2, R2-B3) were used as the detection antigens for all of the positive hybridomas. Based on the result of he ELISA, three MAbs, named 1D6, 1H2, and 3F2, which only recognized RHDV1, were detected (Fig. 1a), and four MAbs, named 1G2, 2C1, 3B7, and 5D6, which only recognized RHDV2, were detected (Fig. 1b). A test of the ability of the MAbs to recognize six short peptides showed that only 1H2 and 3F2 could react with R-A1, which suggested that the antigenic epitope identified by 1H2 and 3F2 was present in R-A1 (amino acids 298–319).

**Figure 2:**
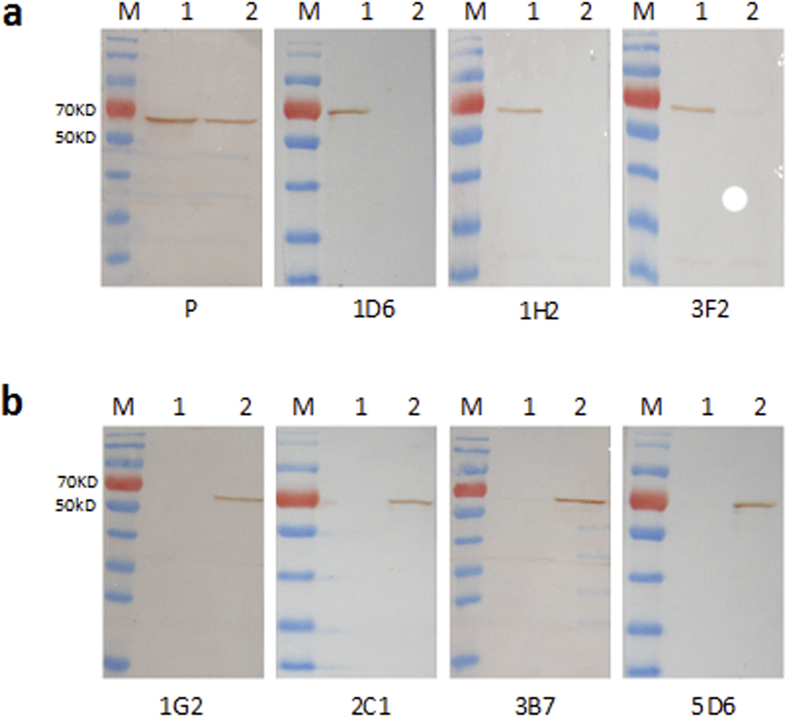
Identification of RHDV type-specific MAbs by Western blotting. After SDS-PAGE, eVP60-1 and eVP60-2, which were expressed in HeLa cells, were transferred to nitrocellulose membranes. MAbs 1D6, 1H2, and 3F2 against RHDV1 (**a**) could only recognize eVP60-1, whereas MAbs 1G2, 2C1, 3B7, and 5D6 against RHDV2 (**b**) could only recognize eVP60-2. M, PageRuler Prestained Protein Ladder; 1, eVP60-1 protein; 2, eVP60-1 protein; P, rabbit hyperimmune serum against RHDV1 was used as a positive control.

**Figure 3:**
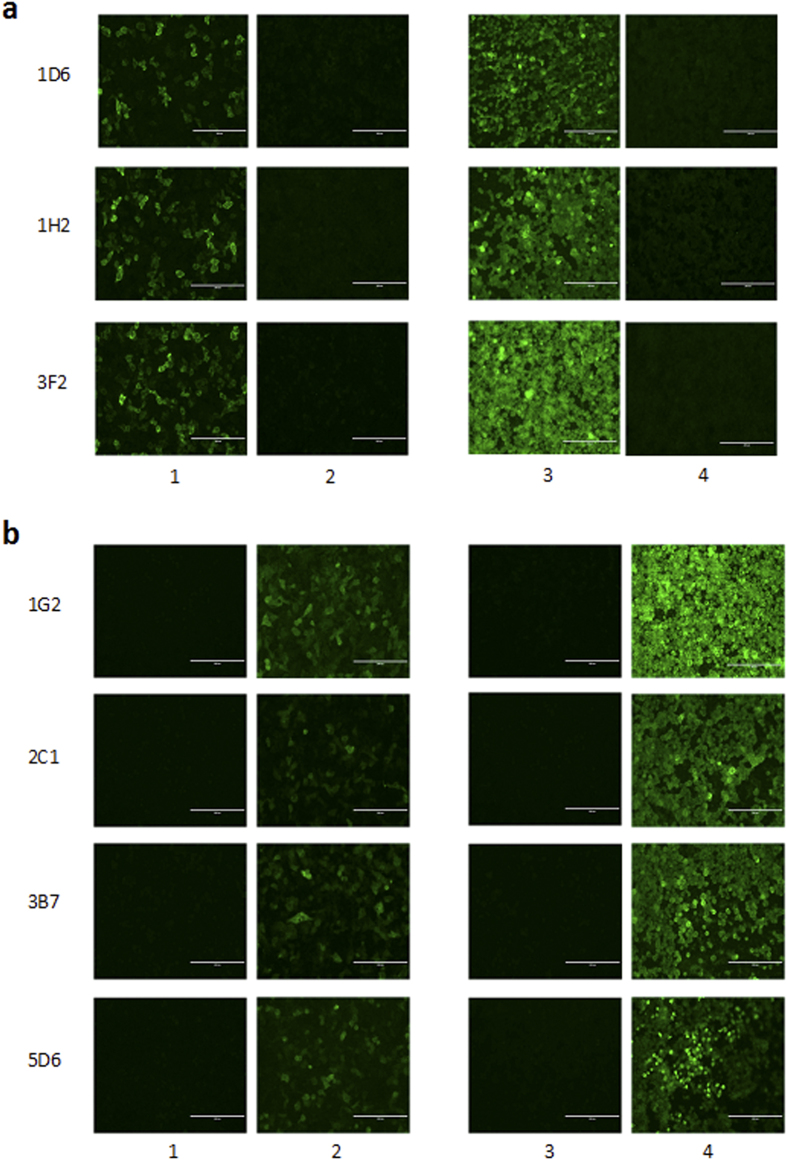
Identification of RHDV type-specific MAbs by IFA. MAbs 1D6, 1H2, and 3F2 against RHDV1 (**a**) only reacted positively with eVP60-1 expressed in HeLa cells and sVP60-1 expressed in Sf9 cells, whereas MAbs 1G2, 2C1, 3B7, and 5D6 against RHDV2 (**b**) only reacted positively with eVP60-2 expressed in HeLa cells and sVP60-2 expressed in Sf9 cells. 1, eVP60-1 expressed in HeLa cells; 2, eVP60-2 expressed in HeLa cells; 3, sVP60-1 expressed in Sf9 cells; 4, sVP60-2 expressed in Sf9 cells.

To further validate the ability of the MAbs to recognize different epitopes, Western blotting and IFA were conducted. For Western blotting, eVP60-1 and eVP60-2 were expressed in HeLa cells and transferred to nitrocellulose membranes after SDS-PAGE. Then, the membranes were reacted with the seven MAbs separately, as well as with rabbit hyperimmune serum against RHDV1 (positive control). Western blotting showed that MAbs 1D6, 1H2, and 3F2 could only recognize eVP60-1 (Fig. 2a), whereas MAbs 1G2, 2C1, 3B7, and 5D6 could only recognize eVP60-2 (Fig. 2b). An IFA performed in Sf9 and HeLa cells also showed that MAbs 1D6, 1H2, and 3F2 only reacted positively with sVP60-1 expressed in Sf9 cells and eVP60-1 expressed in HeLa cells (Fig. 3a), whereas MAbs 1G2, 2C1, 3B7, and 5D6 only reacted positively with sVP60-2 expressed in Sf9 cells and eVP60-2 expressed in HeLa cells (Fig. 3b). The results of the ELISA, Western blotting, and IFA demonstrated that all seven MAbs recognized different features in RHDV1 and RHDV2 and, thus, are type-specific MAbs.

Evaluation of the murine class/subclass of each hybridoma supernatant was conducted using a Pierce® Rapid ELISA Mouse mAb Isotyping Kit (Thermo Fisher Scientific, Waltham, MA, USA). The results showed that 1H2, 3F2, and 2C1 exhibited IgG_1_ isotypes, whereas 1G2, 3B7, and 5D6 exhibited IgG_2a_ isotypes, and 1D6 was classified as IgG_2b_. The light chains of the seven MAbs were all *Kappa* chains (Table 1). The ELISA titers of the MAbs were determined by multiple dilutions of the ascites fluid. Antibody titers in the hydroperitoneum ranged from 2^18^–2^20^ (Table 1).

### Identification of linear B-cell epitopes of RHDV type-specific MAbs

**Figure 4:**
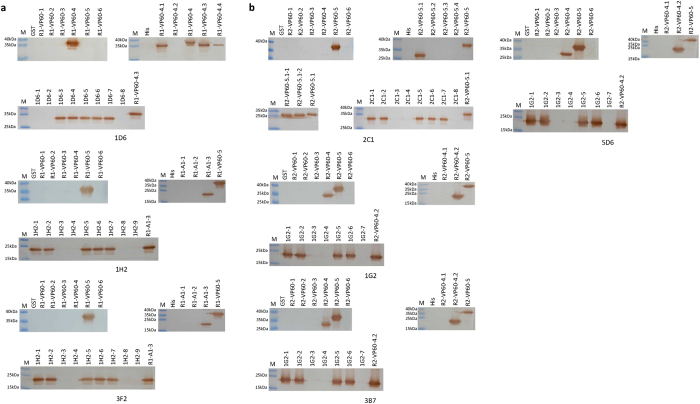
Fine locations of type-specific MAb epitopes. Reactivities of RHDV1 type-specific MAbs (**a**) and RHDV2 type-specific MAbs (**b**) with truncated recombinant VP60 proteins by Western blotting. The names of the truncated proteins are shown in Table 3 and Table 4. A hexahistidine peptide and GST were used as negative controls; the shortest recombinant VP60 protein was used as a positive control. M: PageRuler Prestained Protein Ladder.

His-tagged or glutathione S-transferase (GST)-tagged truncated VP60 proteins were used for fine mapping of the epitopes recognized by the RHDV type-specific MAbs. Western blotting showed that RHDV1 type-specific MAb 1D6 recognized the linear antigenic determinant^256^RWNG***Q***^260^, and MAb 1H2 and 3F2 recognized ^312^VL***QF***W^316^ (amino acids that differ between the RHDV1 and RHDV2 VP60 proteins are identified by italics) (Fig. 4a). The minimal recognition sequence for RHDV2 type-specific MAb 2C1 appeared to be ^324^***A***DNPIS^329^, while MAbs 1G2, 3B7, and 5D6 recognized the sequence ^294^***A***IDH***D***^298^ (Fig. 4b). The results of epitope mapping indicated that 1H2 and 3F2 belonged to one MAb, 1G2, 3B7 and 5D6 also belonged to one MAb as the same. The Western blotting results showed that the epitopes recognized by MAbs 1D6 and 1H2 and 3F2 could react with rabbit hyperimmune serum against RHDV1 (data not shown).

### Sequence analysis of the identified MAb epitopes among RHDV strains

Next, we analyzed the degree of conservation of each identified VP60 epitope among various members of the genus *Lagovirus* in the family *Caliciviridae*. The VP60 amino acid sequence corresponding to each identified linear epitope was identified for representative viruses of RHDV1, RHDV2, RHDVa, rabbit calicivirus (RCV), and European brown hare syndrome virus (EBHSV) using sequences available in the National Center for Biotechnology Information (NCBI) database. The amino acid sequence for each identified RHDV VP60 epitope was then aligned with the corresponding sequences from each of the different lagoviruses using MegAlign software. The result showed that the RHDV1 VP60 epitopes that are recognized by different MAbs are highly conserved (98%) among RHDV1 strains, whereas the RHDV2 VP60 epitopes that are recognized by different MAbs are completely conserved (100%) among RHDV2 strains (Table 2). Residue ^260^Q in the epitope recognized by MAb 1D6 and residues ^314^Q and ^315^F in the epitope recognized by MAbs 1H2 and 3F2 were completely conserved among all the RHDV1 strains selected in this study, whereas the corresponding residues in all of the RHDV2 strains were glutamic acid, glutamic acid, and leucine, respectively. Additionally, the ^324^A residue in the epitope recognized by MAb 2C1 and the ^294^A and ^298^D residues in the epitope recognized by MAbs 1G2, 3B7, and 5D6 were completely conserved among all the RHDV2 strains, whereas the corresponding residues in the RHDV1 strains were isoleucine or valine, aspartic acid, and arginine, respectively. Thus, these amino acids are key residues that account for the epitope recognition differences of the MAbs.

According to the results of the sequence alignment, the corresponding sequences of the RHDV1 HYD strain (GenBank accession no.: AEB26305.1) and the RHDV1 Meiningen strain (GenBank accession no.: CAA75624.1) have a single amino acid difference compared with the other RHDV1 strains (asparagine instead of aspartic acid at position 258 and valine instead of glycine at position 259, respectively) in the epitope recognized by MAb 1D6, while the RHDV1 HYD strain also has a single amino acid difference in the epitope recognized by MAbs 1H2 and 3F2 (alanine instead of valine at position 312). Next, we expressed these corresponding peptides (^256^RWDGQ^260^, ^256^RWNVQ^260^ and ^312^ALQFW^316^) by prokaryotic, and the Western blotting results showed that these corresponding peptides could react with 1D6 or 1H2 and 3F2 accordingly (data not shown). These results indicate that the ability of the screened MAbs to recognize different epitopes was quite stable, and that they might be able to distinguish different subtypes of RHDV; thus, they are possibly type-specific MAbs.

## Discussion

Despite an extensive vaccination and outbreak control strategy, RHDV is still a major economical and ecological burden in many parts of the world^25^. Until recently, only one serotype was circulating^26^; however, it seems possible that a new serotype (termed RHDVb or RHDV2) has emerged^14^,^15^,^17^,^19^. In terms of its phylogenetic position, antigenic profile, and pathogenicity, RHDV2, which emerged from RHDV1, may be a new member of the *Lagovirus* genus^17^. Significantly, a RHDV1 vaccine has been reported to have low or no efficacy against RHDV2^13^,^15^. RHDV2 has spread quickly and appears to be replacing older RHDV1 strains on the Iberian Peninsula and in France, and it has also spread to Italy and the United Kingdom^13^,^15^,^17^,^21^. To date, RHDV2 has not been detected in China. The non-enveloped RHDV virus capsid is constructed from the unique structural protein VP60, which is a major determinant of viral pathogenicity and antigenicity. The complete recombinant VP60 protein has been shown to be antigenic when expressed from different constructs, and to induce a detectable protective response^27^,^28^,^29^,^30^,^31^. VP60 is the main target for new vaccines, as well as for the development of immunity-based prophylactic, therapeutic, and diagnostic techniques for controlling RHDV. In this study, we produced seven RHDV type-specific MAbs and discovered four linear B-cell epitopes at the carboxyl-terminus of VP60.

The VP60-specific MAbs in this study were produced using recombinant VP60 proteins from different RHDV subtypes that were expressed in a prokaryotic expression system. Twenty of the most reactive MAbs against RHDV1 and 15 MAbs against RHDV2 were selected based on the ELISA results. The RHDV TP strain, pVP60-1, pVP60-2, and six short peptides were used as the detection antigens in indirect ELISAs with all of the positive hybridomas to determine whether the screened MAbs were type-specific. Finally, three MAbs (1D6, 1H2, and 3F2) that only recognized RHDV1 were detected, while four MAbs (1G2, 2C1, 3B7, and 5D6) that only recognized RHDV2 were detected. The Western blotting and IFA results further validated the differences in epitope recognition of these MAbs. These seven MAbs were all IgG isotypes, and their antibody titers were quite high.

According to the Western blotting results, all seven MAbs positively reacted with denatured recombinant VP60, suggesting that their antigenic recognition sites were linear epitopes. To study their specificity in more detail and to map the epitopes bound by the MAbs, we expressed a series of partially overlapping His- or GST-tagged fragments of the VP60 proteins of the RHDV1 TP strain or RHDV2 10–32 strain and used them to screen for the minimal epitope via Western blotting. This identified the minimal sequence of the MAb 1D6-defined epitope as ^256^RWNG***Q***^260^, while MAbs 1H2 and 3F2 recognized the sequence ^312^VL***QF***W^316^ (amino acids that differ between the RHDV1 and RHDV2 VP60 proteins are identified by italics), whereas MAb 2C1 recognized the linear antigen ^324^***A***DNPIS^329^, and MAbs 1G2, 3B7, and 5D6 recognized the sequence ^294^***A***IDH***D***^298^; the deletion of any residues from either end of these core sequences inhibited binding by the corresponding MAbs. It has been reported that one MAb 1G5, which recognizes a linear epitope with the sequence: NPISQVAP (amino acids positions 326–333 of VP60 protein) located at loop L2 of P2 subdomain, and reacted with both RHDV1 and RHDV2 capsid proteins^18^. Comparing the epitope of MAb1G5 with MAb 2C1in our study, it showed that ^324^A is the key amino acids that affect the epitope recognition ability of the MAb 2C1. Identification of epitopes showed that MAbs 1H2 and 3F2 recognized the same epitope ^312^VLQFW^316^ and MAbs 1G2, 3B7 and 5D6 also recognized the same epitope ^294^AIDHD^298^. However MAbs 1H2 and 3F2 of RHDV1 originated from different hybridoma culture wells, and it was similar to MAbs 1G2, 3B7 and 5D6 of RHDV2, which means ^312^VLQFW^31^ and ^294^AIDHD^298^ might be immunodominant epitopes. The results of epitope mapping indicated that 1H2 and 3F2 belonged to one MAb, 1G2, 3B7 and 5D6 also belonged to one MAb as the same. Previous reports grouped RHDV epitopes into three different types based on Western blotting and/or ELISA reactivities: surface linear, surface conformational, and internal linear epitopes^32^,^33^,^34^. The recognition abilities of these MAbs could indicate that they bind surface linear epitopes. The most exposed P2 sub-domain showed the highest degree of genetic variation^11^, and it may contain an important epitope for anti-RHDV antibody production. However, the previously reported epitopes of RHDV were all located in the most amino-terminal or carboxyl-terminal regions of the capsid proteins^32^,^33^,^35^,^36^,^37^. In this study, all seven MAb epitopes were located in the P2 sub-domain, except for that of MAb 1D6, which was located in the P1 sub-domain. To the best of our knowledge, this represents the first fine mapping of B-cell epitopes in the P2 sub-domain of VP60.

An analysis to assess the conservation of the identified MAb epitopes among members of the genus *Lagovirus* was performed. The results showed that the RHDV1 VP60 epitopes recognized by the MAbs are highly conserved (98%) among RHDV1 strains, whereas the RHDV2 VP60 epitopes recognized by the MAbs are completely conserved (100%) among RHDV2 strains. Furthermore, MAb 1D6 and MAbs 1H2 and 3F2 could also react with the RHDV1 TP strain and the RHDV1 Meiningen strain, which exhibited single amino acid differences according to the results of the sequence alignment. In conclusion, the ability of the screened MAbs to recognize different epitopes was quite stable, and they might be able to distinguish different subtypes of RHDV; thus, they are possibly type-specific MAbs. The key amino acid residues that account for the recognition differences of the MAbs are all completely conserved. Previous results showed that RHDV2 differs from RHDV1 in terms of its disease duration, mortality rates, higher occurrence of subacute/chronic forms, and antigenic profile^17^,^18^. Amino acids that affect the epitope recognition ability of the type-specific MAbs may also be the key residues that determine the antigenic differences between RHDV1 and RHDV2.

This study prepared RHDV type-specific MAbs by hybridoma fusion and precisely mapped the epitopes that they recognize. Type-specific MAb preparation provides the foundation for the establishment of RHDV subtype-specific detection methods, and it has important significance for RHDV epidemiological investigations and phylogenetic analyses. The established MAbs and defined epitopes could be used to further study the structure and function of VP60 during RHDV infection and to provide a foundation for the development of novel epitope-based vaccines. Furthermore, the truncated, recombinant proteins generated in this study may by useful tools for mapping other epitopes on RHDV VP60 proteins in future studies.

## Methods

### Expression of recombinant VP60

The RHDV1 TP strain (GenBank accession no.: AF453761.1) was acquired from the State Key Laboratory of Veterinary Biotechnology, Harbin Veterinary Research Institute, Chinese Academy of Agricultural Sciences (CAAS). The *VP60* gene of the RHDV1 TP strain was previously cloned into the pGEX-6P-1 vector, and the recombinant plasmid was named pGEX-R1-VP60^38^. The *VP60* gene of the RDHV2 10–32 strain (GenBank accession no.: HE800532.1) was synthesized (Harbin Boyuan Biological Technology Co., Ltd., Harbin, China) and cloned into the pMD-18T vector to yield pMD-R2-VP60. The *VP60* genes of RHDV1 and RHDV2 were amplified from pGEX-R1-VP60 and pMD-R2-VP60, respectively, following agarose gel purification and restriction enzyme digestion. Genes were cloned into the vector pET-32a using the EcoRI and HindIII sites introduced into the sense and antisense primers, respectively. The sequences and locations of the primers are shown in Table 3 and Table 4. The recombinant plasmids were verified by restriction enzyme digestion and DNA sequencing (Harbin Boyuan Biological Technology Co., Ltd.) and then transformed into *E. coli* BL21 Star(DE3)pLysS cells (Biovector Science Lab, Inc, China) to produce recombinant, his-tagged VP60. Specifically, the cells were shaken for several hours until they reached an optical density at 600 nm of 0.8–1.0, at which point IPTG (IPTG, Solarbio, China)was added at a final concentration of 0.05 mM to Luria-Bertani (LB) medium (each liter included 10 g of tryptone, 5 g of yeast extract, 10 g of NaCl, sterile ampicillin to 500 μg/ml, and sterile chloromycetin to 340 μg/ml), followed by a further 18-h incubation at 16 °C with shaking. Then, the bacteria were pelleted by centrifugation at 12,000 × *g* for 10 min at 4 °C and resuspended in one-tenth volume (relative to the original culture volume) of phosphate-buffered saline (PBS, pH 7.4). SDS-PAGE was conducted to characterize the production of His-tagged recombinant VP60 proteins from different RHDV subtypes.

The recombinant VP60 proteins from the different RHDV subtypes were purified from a preparative (1.5 mm) SDS-PAGE gel after staining with 0.25 mM KCl^39^. Briefly, the recombinant VP60 proteins from the different RHDV subtypes were expressed in *E. coli* BL21 Star(DE3)pLysS cells, and they were separated using SDS-PAGE. The target proteins were isolated by cutting out the gel slices containing the appropriate bands, which were stained by 0.25 mM KCl. After washing three times with PBS (pH 7.4), the gel slices were transferred to a clean bag and crushed. An appropriate volume of PBS was added, and the gel pieces were subjected to three cycles of freezing and thawing at −20 °C to release the proteins. Finally, the supernatant was collected by centrifugation at 12,000 × *g* for 2 min, and the extracted VP60 proteins from the different RHDV subtypes, named pVP60-1 and pVP60-2, were used as an immunogens after antigenicity testing by Western blotting. Rabbit hyperimmune serum against RHDV1 (generated in our laboratory) was used as the primary antibody for Western blotting as described below.

VP60 was also expressed in two eukaryotic expression systems. In the first system, a recombinant RHDV2 *VP60* baculovirus was constructed, and the VP60 proteins of the different RHDV subtypes were expressed in insect cells using the Bac-to-Bac Baculovirus Expression System (Invitrogen, Carlsbad, CA, USA) as previously described^31^. sVP60-1 and sVP60-2 were used for IFA analysis of the screened MAbs. In the second system, the *VP60* genes of RHDV1 and RHDV2 were cloned into the eukaryotic expression vector pcDNA3.1(+) (Invitrogen, USA) and expressed in HeLa cells. Briefly, the *VP60* genes of RHDV1 and RHDV2 were amplified from plasmids pGEX-R1-VP60 and pMD-R2-VP60, respectively, by polymerase chain reactions (PCRs) with the primers shown in Table 3 and Table 4. The PCR products were separately cloned into the vector pcDNA3.1(+) at the Hind III and EcoRI sites, respectively, according to the manufacturer’s instructions. The recombinant plasmids pcDNA-R1-VP60 and pcDNA-R2-VP60 were verified by restriction enzyme digestion and DNA sequencing (Harbin Boyuan Biological Technology Co., Ltd.) and then separately transfected into HeLa cells using Lipofectamine^TM^ 2000 Reagent (Invitrogen) according to the manufacturer’s protocol. The RHDV1 and RHDV2 VP60 proteins that were expressed in HeLa cells were named eVP60-1 and eVP60-2, respectively. Forty-eight hours after transfection, cells were fixed with cold 75% ethanol for IFA, or the cell lysis supernatant was collected for Western blotting analysis. The expression of the recombinant VP60 proteins in Sf9 and HeLa cells was confirmed by IFA as described below.

### Immunization protocol and hybridoma production

The purified VP60 proteins from different RHDV subtypes were diluted to 500 μg/ml with PBS (pH 7.4). Adult female BALB/c mice (Division of Laboratory Animal Research Center, Harbin Veterinary Research Institute), 6–8 weeks of age, were divided into two groups, and their hind leg muscles were injected intramuscularly with 100 μl of the purified VP60 proteins from the different RHDV subtypes, which was mixed at a 1:1 (v/v) ratio with the QuickAntibody™ Adjuvant (Beijing Biodragon Immunotechnologies Co., Ltd., Beijing, China), thus yielding a total injection volume of 200 μl. Injections were given on days 7 and 21. One week after the last boost and 3 days before fusion with myeloma cells, the mice were re-challenged intramuscularly with 100 μg of the antigen in sterile PBS (pH 7.4).

Three days after intravenous injection, serum antibody titers were monitored by ELISA, and high-titer antibody-producing animals were sacrificed for hybridoma production. Single splenocyte suspensions were prepared from the spleen of a selected mouse and fused with SP2/0 myeloma cells at a ratio of 5:1 using 50% polyethylene glycol (50% PEG, Sigma-Aldrich, St. Louis, MO, USA). Hybridoma cells were suspended in hypoxanthine-aminopterin-thymidine (HAT, Sigma-Aldrich) selection medium (Dulbecco’s modified Eagle’s medium (DMEM, Gibco, Grand Island, NY, USA) containing 20% fetal bovine serum, 100 mg/ml streptomycin, 100 IU/ml penicillin, 100 mM hypoxanthine, 16 mM thymidine, and 400 mM aminopterin). The resulting cell suspension was distributed in 96-well culture plates containing 1 × 10^5^ cells/well. Plates were incubated in a 5% CO_2_ atmosphere at 37 °C. Half of the medium from each well was removed and HT medium (HAT lacking aminopterin) (Sigma-Aldrich, USA) was added on days 3 and 8. Wells that contained growing cells (positive wells) were screened for antibody production by testing supernatants in a standard indirect ELISA using *E. coli* that expressd the VP60 proteins of the corresponding subtypes. Positive clones were subcloned by limiting dilution. After three rounds of subcloning, the positive clones were further confirmed by an indirect ELISA, Western blotting, and an IFA, as described below. The animal experiments were approved by the Animal Ethics Committee of Harbin Veterinary Research Institute (HVRI) of the Chinese Academy of Agricultural Sciences and performed in accordance with animal ethics guidelines and approved protocols. The Animal Ethics Committee approval number was SYXK (Hei) 2011–022.

### ELISAs

The purified VP60 proteins from the different RHDV subtypes were diluted to 6 μg/ml with carbonate buffer solution (0.015 M Na_2_CO_3_, 0.034 M NaHCO_3_, pH 9.6). Ninety-six-well microtiter ELISA plates were separately coated with the VP60 proteins (100 μl/well) overnight at 4 °C. The plates were washed three times with PBS containing 0.05% Tween-20 (PBST), and then blocked with 250 μl/well of 5% skim milk powder in PBST for 3 h at 37 °C. One hundred microliters of supernatant fluid from each hybridoma was added to each well and incubated for 1 h at 37 °C. After washing four times with PBST, the plates were incubated with a 1:80,000 dilution of horseradish peroxidase (HRP)-conjugated goat anti-mouse IgG for 1 h at 37 °C. Following the incubation, the plates were washed three times with PBST, and a chromogenic reagent (3,3′,5,5′-tetramethylbenzidine (TMB); Sigma-Aldrich, USA) was added. The absorbance was measured at 450 nm in a microplate reader (Bio Tek, USA).

### Identification of MAbs targeting different RHDV subtypes

After three rounds of subcloning, positive hybridoma clones of RHDV1 and RHDV2 were identified by an indirect ELISA to determine whether the screened MAbs were type-specific. Based on a comparison of the RHDV1 and RHDV2 VP60 amino acid sequences, six short peptides, which had subtype-specific sequence differences, were synthesized (Table 5). The RHDV TP strain (2^7^ hemagglutination (HA) units), pVP60-1 (600 ng/well), pVP60-2 (600 ng/well), and the six short peptides (500 ng/well) were separately coated onto an ELISA plate and used as the detection antigens in an indirect ELISA with all of the positive hybridomas mentioned above. The results were compared among the different groups by one-way repeated measures analysis of variance (ANOVA) using SAS 9.1 statistical software. Differences among the groups were considered to be statistically significant at *P* < 0.05 (n = 4). Based on the results of the indirect ELISA, the preliminary screening type-specific MAbs were further determined by Western blotting and IFA.

The VP60 proteins from RHDV1 and RHDV2 were individually expressed in HeLa cells 48 h after transfection. Cells were lysed in lysis buffer (150 mM Tris-HCl (pH 7.6), 50 mM NaCl, 5 mM ethylenediaminetetraacetic acid (EDTA), 1% Triton-X100). The cell lysates were heated at 100 °C for 10 min after the addition of 5× SDS-PAGE loading buffer (250 mM Tris-HCl, pH 6.8, 10% SDS, 0.5% bromophenol blue, 50% glycerol, 5% β-mercaptoethanol), and the proteins were separated by SDS-PAGE on a 12% polyacrylamide gel. After electrophoresis, the separated proteins were electrophoretically transferred to a nitrocellulose membrane (Pall Life Sciences, Port Washington, NY, USA) at 10 V for 60 min using a Pierce G2 Fast Blotter (Thermo Fisher Scientific, USA). After the transfer, the membranes were blocked overnight at 4 °C in TBST containing 5% skim milk. The blots were washed three times in TBS containing 0.05% Tween-20 (TBST) at the end of each step, and then incubated at 37 °C with a type-specific hybridoma supernatant for 1 h. After washing, the membranes were sequentially incubated with a 1:2,000 dilution of HRP-goat anti-mouse IgG in 5% skim milk for 1 h at 37 °C. After the final wash, the bands were visualized using 3,3′-diaminobenzidine-peroxidase (DAB, Sigma-Aldrich, USA) as a substrate. The reaction was stopped by rinsing the membrane with distilled water.

IFA experiments were performed using Sf9 cells cultured in 96-well plates and HeLa cells cultured in 48-well plates. Sf9 cells were separately infected with a recombinant RHDV1 *VP60* baculovirus^31^ and a recombinant RHDV2 *VP60* baculovirus. HeLa cells were individually transfected with pcDNA-R1-VP60 and pcDNA-R2-VP60. After the infections or transfections, the cells were fixed with cold 75% ethanol for 10 min at 4 °C. Then, the fixed cells were incubated with a type-specific hybridoma supernatant or the SP2/0 supernatant (negative control) for 1 h at 37 °C. Fluorescein isothiocyanate (FITC)-conjugated goat anti-mouse IgG was used as a secondary antibody at a 1:100 dilution, and plates were viewed at a magnification of 20× using a fluorescence microscope (AMG, USA).

### Characterization of type-specific MAbs

The isotypes of the type-specific MAbs were determined using the Pierce® Rapid ELISA Mouse mAb Isotyping Kit (Thermo Fisher Scientific, USA) according to the manufacturer’s protocol. The inducing ascites *in vivo* method was employed to produce large amounts of the MAbs^40^. Ascites fluids of type-specific MAb titers were monitored by indirect ELISA as previously described.

### Epitope mapping

The epitopes of type-specific MAbs were then defined by Western blotting. Linear epitopes of VP60 were predicted using the Immune Epitope Database (IEDB http://www.iedb.org/). Based on the predictions, six truncated polypeptides were designed (Table 5). All truncated *VP60* genes of the different RHDV subtypes were amplified from pGEX-R1-VP60 or pMD-R2-VP60, and the gene fragments were cloned into the pET-32a or pGEX-6p-1 vectors. The sequences and locations of the primers are shown in Table 3 and Table 4. Recombinant truncated VP60 polypeptides were expressed and purified as described above. Purified, recombinant truncated VP60 proteins were separated by 12% SDS-PAGE. For Western blotting, the proteins were transferred onto a nitrocellulose membrane and detected as previously described. Purified His- and GST-tagged proteins were used as negative antigen controls.

### Sequence analysis of the identified linear peptide epitopes among RHDV strains

An analysis to assess the conservation of the identified MAb epitopes among members of the genus *Lagovirus* was performed. Amino acid sequences corresponding to the identified type-specific MAb epitope regions of various lagoviruses, including different RHDV1 and RHDV2 isolates, as well as RHDVa, RCV, and EBHSV reference strains, were aligned using MegAlign bioinformatics software (Lasergene, DNASTAR Inc., Madison, WI, USA). The sequences used are all available in the NCBI database (http://www.ncbi.nlm.nih.gov/protein/). All experimental protocols were approved by Basic Scientific Research Operation Cost of State-leveled Public Welfare Scientific Research Courtyard (0302015002).

## Additional Information

**How to cite this article**: Kong, D. *et al.* Production, Characterization, and Epitope Mapping of Monoclonal Antibodies Against Different Subtypes of Rabbit Hemorrhagic Disease Virus (RHDV). *Sci. Rep.* **6**, 20857; doi: 10.1038/srep20857 (2016).

## Supplementary Material

Supplementary Information

## Figures and Tables

**Table 1:** Isotypes and hydroperitoneum antibody titers of representative type-specific clones.

MAb	MAbs strain	Isotype	ELISA titers	MAb	MAbs strain	Isotype	ELISA titers
1D6	RHDV1	IgG_2b_, *k*	2^18^	1G2	RHDV2	IgG_2a_, *k*	2^18^
1H2	RHDV1	IgG_1_, *k*	2^20^	2C1	RHDV2	IgG_1_, *k*	2^18^
3F2	RHDV1	IgG_1_, *k*	2^20^	3B7	RHDV2	IgG_2a_, *k*	2^18^
				5D6	RHDV2	IgG_2a_, *k*	2^18^

**Table 2:** Sequences analysis of the MAb antigenic epitopes of different RHDV subtypes.

Virus strain	Accession number	MAb 1D6	MAb 1H2/3F2	MAb 2C1	MAb 1G2/3B7/5D6
RHDV1	AAM21587.1	^256^RWNGQ^260^	^312^VLQFW^316^	**I** DNPIS	**D**IDH**R**
RHDV1	AAK85434.1	RWNGQ	VLQFW	**I** DNPIS	**D**IDH**R**
RHDV1	AAP15339.1	RWNGQ	VLQFW	**I** DNPIS	**D**IDH**R**
RHDV1	ABA46867.1	RWNGQ	VLQFW	**I** DNPIS	**D**IDH**R**
RHDV1	ABH10017.1	RWNGQ	VLQFW	**I** DNPIS	**D**IDH**R**
RHDV1	ACB28856.1	RWNGQ	VLQFW	**I** DNPIS	**D**IDH**R**
RHDV1	AEB26305.1	RW**D**GQ	**A**LQFW	**I** DNPIS	**D**IDH**R**
RHDV1	AEU09705.1	RWNGQ	VLQFW	**I** DNPIS	**D**IDH**R**
RHDV1	AEU09706.1	RWNGQ	VLQFW	**V**DNPIS	**D**IDH**R**
RHDV1	AEU09707.1	RWNGQ	VLQFW	**I** DNPIS	**D**IDH**R**
RHDV1	AEU09708.1	RWNGQ	VLQFW	**I** DNPIS	**D**IDH**R**
RHDV1	AFN69440.1	RWNGQ	VLQFW	**I** DNPIS	**D**IDH**R**
RHDV1	CAA75624.1	RWN**V**Q	VLQFW	**V**DNPIS	**D**IDH**R**
RHDV1	CAA80883.1	RWNGQ	VLQFW	**I** DNPIS	**D**IGH**R**
RHDV1	CAA75625.1	RWNGQ	VLQFW	**I** DNPIS	**D**IDH**R**
RHDV1	CAA75631.1	RWNGQ	VLQFW	**I** DNPIS	**D**IDH**R**
RHDV1	CAA75632.1	RWNGQ	VLQFW	**I** DNPIS	**D**IDH**R**
RHDV1	CAA75633.1	RWNGQ	VLQFW	**I** DNPIS	**D**IDH**R**
RHDV1	CAD91718.1	RWNGQ	VLQFW	**I** DNPIS	**D**IDH**R**
RHDV1	NP_740333.1	RWNGQ	VLQFW	**I** DNPIS	**D**IDH**R**
RHDV1	CAA60910.1	RWNGQ	VLQFW	**V**DNPIS	**D**IDH**R**
RHDVa	ABX38989.2	RWNGQ	VLQFW	**I** DNPIS	**D**IDH**R**
RCV	CAA65611.1	RWN**C**Q	VLQFW	**V**DNPIC	**D**IDH**R**
EBHSV	CAA66639.1	RW**GAP**	**T** **I** **ET**W	**T T**NPIS	**D**IDH**R**
RHDV2	CCH15347.1	RWNG**E**	VL**EL**W	^324^ADNPIS^329^	^294^AIDHD^298^
RHDV2	CCH15344.1	RWNG**E**	VL**EL**W	ADNPIS	AIDHD
RHDV2	CCH15345.1	RWNG**E**	VL**EL**W	ADNPIS	AIDHD
RHDV2	CCH15346.1	RWNG**E**	VL**EL**W	ADNPIS	AIDHD
RHDV2	CCH80663.1	RWNG**E**	VL**EL**W	ADNPIS	AIDHD
RHDV2	CBZ39415.3	RWNG**E**	VL**EL**W	ADNPIS	AIDHD
RHDV2	AGC11803.1	RWNG**E**	VL**EL**W	ADNPIS	AIDHD
RHDV2	AGC11805.1	RWNG**E**	VL**EL**W	ADNPIS	AIDHD
RHDV2	AGW27412.1	RWNG**E**	VL**EL**W	ADNPIS	AIDHD
RHDV2	AGW27413.1	RWNG**E**	VL**EL**W	ADNPIS	AIDHD
RHDV2	AGW27414.1	RWNG**E**	VL**EL**W	ADNPIS	AIDHD
RHDV2	AHL89009.1	RWNG**E**	VL**EL**W	ADNPIS	AIDHD

**Table 3:** Sequences of the primers and vectors used for RHDV1 type-specific MAb epitope identification.

Fragment	Primer sequences (5′–3′)^A^		Position In VP60 gene^B^	Vector
	Sense	Negative –sense		
pR1-VP60	GGAATTCATGGAGGGCAAAACCCGCAC	CCCAAGCTTGTCAGACATAAGAAAAGCCATTGGT	1–1740	pET-32a
eR1-VP60	CCCAAGCTTATGGAGGGCAAAACCCGCAC	GGAATTCTCAGACATA AGA AAAGCCATTGGT	1–1740	pcDNA3.1(+)
R1-VP60-1	GGAATTCATGGAGGGCAAAACCCGCAC	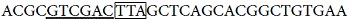	1–342	pGEX-6p-1
R1-VP60-2	GGAATTCCCATTCACAGCCGTGCTGAG	CCCAAGCTTGTAGTGTGGGGACAAGGCCAG	301–570	pET-32a
R1-VP60-3	GGAATTCGACTTGCGTCCCAACAT	CCCAAGCTTGTGCGGGTGAGATTGAGT	514–720	pET-32a
R1-VP60-4	GGAATTCAGAGCCCCCTCCAGCAAGAC	CCCAAGCTTGGCCTCTTCGATGGTCAATGTC	679–900	pET-32a
R1-VP60-5	GGAATTCTGGTCAAGCCCTCGGTTTGC	CCCAAGCTTGAGCAGGAGTGCCGGGTGTAG	859–1353	pET-32a
R1-VP60-6	GGAATTCGTCACATACACGCCCCAAC	CCCAAGCTTGTCAGACATAAGAAAAGCCATTGGT	1300–1740	pET-32a
R1-VP60-4.1	GGAATTC CCCGCAGGCCTCCTCACGAC	CCCAAGCTTGTTGCAGTCCCACTATTTGGC	715–795	pET-32a
R1-VP60-4.2	GGAATTCATAGTGGGACTGCAACCAGT	CCCAAGCTTGCCGAGGGCTTGACCAGCCAT	781–873	pET-32a
R1-VP60-4.3	GGAATTCCCCGCAGGCCTCCTCACGAC	CCCAAGCTTGGGAAAACCCCCCAGGTACTG	715–816	pET-32a
R1-VP60-4.4	GGAATTCAGGTGGAACGGCCAAATAGT	CCCAAGCTTGCCGAGGGCTTGACCAGCCAT	766–873	pET-32a
1D6-1	GGAATTCTGGAACGGCCAAATAGTGGGACTGCAA	CCCAAGCTTGTTGCAGTCCCACTATTTGGCCGTTCCA	769–795	pET-32a
1D6-2	GGAATTCAACGGCCAAATAGTGGGACTGCAA	CCCAAGCTTGTTGCAGTCCCACTATTTGGCCGTT	771–795	pET-32a
1D6-3	GGAATTCAGGTGGAACGGCCAAATAGTGGGACTG	CCCAAGCTTGCAGTCCCACTATTTGGCCGTTCCACCT	766–792	pET-32a
1D6-4	GGAATTCAGGTGGAACGGCCAAATAGTGGGA	CCCAAGCTTGTCCCACTATTTGGCCGTTCCACCT	766–789	pET-32a
1D6-5	GGAATTCAGGTGGAACGGCCAAATAGTG	CCCAAGCTTGCACTATTTGGCCGTTCCACCT	766–786	pET-32a
1D6-6	GGAATTCAGGTGGAACGGCCAAATA	CCCAAGCTTGTATTTGGCCGTTCCACCT	766–783	pET-32a
1D6-7	GGAATTCAGGTGGAACGGCCAA	CCCAAGCTTGTTGGCCGTTCCACCT	766–780	pET-32a
1D6-8	GGAATTCAGGTGGAACGGC	CCCAAGCTTGGCCGTTCCACCT	766–777	pET-32a
R1-A1-1	GGAATTCCGAAGAGGCAGTGCAAGTTATTCTGGGAACAACTCC	CCCAAGCTTGGGAGTTGTTCCCAGAATAACTTGCACTGCCTCTTCG	892–927	pET-32a
R1-A1-2	GGAATTCTCTGGGAACAACTCCACCAACGTGCTCCAG	CCCAAGCTTGCTGGAGCACGTTGGTGGAGTTGTTCCCAGA	913–942	pET-32a
R1-A1-3	GGAATTCACCAACGTGCTCCAGTTTTGGTACGCTAAT	CCCAAGCTTGATTAGCGTACCAAAACTGGAGCACGTTGGT	928–957	pET-32a
1H2-1	GGAATTCAACGTGCTCCAGTTTTGGTACGCTAAT	CCCAAGCTTGATTAGCGTACCAAAACTGGAGCACGTT	931–957	pET-32a
1H2-2	GGAATTCGTGCTCCAGTTTTGGTACGCTAAT	CCCAAGCTTGATTAGCGTACCAAAACTGGAGCAC	934–957	pET-32a
1H2-3	GGAATTCCTCCAGTTTTGGTACGCTAAT	CCCAAGCTTGATTAGCGTACCAAAACTGGAG	937–957	pET-32a
1H2-4	GGAATTCCAGTTTTGGTACGCTAAT	CCCAAGCTTGATTAGCGTACCAAAACTG	940–957	pET-32a
1H2-5	GGAATTCACCAACGTGCTCCAGTTTTGGTACGCT	CCCAAGCTTGAGCGTACCAAAACTGGAGCACGTTGGT	928–954	pET-32a
1H2-6	GGAATTCACCAACGTGCTCCAGTTTTGGTAC	CCCAAGCTTGGTACCAAAACTGGAGCACGTTGGT	928–951	pET-32a
1H2-7	GGAATTCACCAACGTGCTCCAGTTTTGG	CCCAAGCTTGCCAAAACTGGAGCACGTTGGT	928–948	pET-32a
1H2-8	GGAATTCACCAACGTGCTCCAGTTT	CCCAAGCTTGAAACTGGAGCACGTTGGT	928–945	pET-32a
1H2-9	GGAATTCACCAACGTGCTCCAG	CCCAAGCTTGCTGGAGCACGTTGGT	928–942	pET-32a

^A^Underlined letters indicate additional restriction enzyme sites: EcoRI and Hind III for the vector pET-32a, EcoRI and SalI for the vector pGEX-6P-1, and HindIII and EcoRI for the vector pcDNA3.1(+) were introduced into each primer. The boxed TTAs are the stop codon additions introduced into the antisense primers of the VP60 fragments for use with pGEX-6P-1. ^B^Nucleotide positions correspond to those in the sequence of the RHDV1 *VP60* gene (GenBank accession no.: AF453761.1).

**Table 4:** Sequences of the primers and vectors used for RHDV2 type-specific MAbs epitope identification.

Fragment	Primer sequences (5′-3′)^A^		Position In VP60 gene^B^	Vector
	Sense	Negative –sense		
pR2-VP60	GGAATTCATGGAGGGCAAAGCCCGCGC	CCCAAGCTTGTCAGACATAAGAAAAGCCATTAGT	1–1740	pET-32a
eR2-VP60	CCCAAGCTTATGGAGGGCAAAGCCCGCGC	GGAATTCTCAGACATA AGAAAAGCCATTAGT	1–1740	pcDNA3.1(+)
R2-VP60-1	GGAATTCATGGAGGGCAAAGCCCGCGC	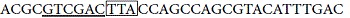	1–339	pGEX-6p-1
R2-VP60-2	GGAATTCCACTCGCCACAAAACAATCC	CCCAAGCTTGCAACGTGGGAACGAGGCCAG	283–570	pET-32a
R2-VP60-3	GGAATTCCCGGACTTGCGCCCCAACAT	CCCAAGCTTGTGCGGGCGAGATCGAGTCAA	511–720	pET-32a
R2-VP60-4	GGAATTC GTGATGATCCGTGCCCCCTC	CCCAAGCTTGACCTTTGTCGTGGTCAATGG	670–900	pET-32a
R2-VP60-5	GGAATTCTGGTCAAGCCCGCGGTTTGC	CCCAAGCTTGAGCAGGGGTACCAGGTGCAT	859–1353	pET-32a
R2-VP60-6	GGAATTCATCACGTACACACCCCAGCC	CCCAAGCTTGTCAGACATAAGAAAAGCCATTAGT	1300–1740	pET-32a
R2-VP60-5.1	GGAATTCGGTAAAGCAAGTTACCCTGGA	CCCAAGCTTGGGGTACAAATGACATGTCAGG	898–1029	pET-32a
R2-VP60-5.2	GGAATTCTCCCAAATTGCTCCAGATGGT	CCCAAGCTTGTAACTCATAAGCCTGCACGG	985–1134	pET-32a
R2-VP60-5.3	GGAATTCAGCAATAATGGTGCCCCCTT	CCCAAGCTTGCAACCCGGCTGTTGCCTGGT	1087–1254	pET-32a
R2-VP60-5.4	GGAATTCATCTATGGCGTTGCAACTGG	CCCAAGCTTGCGTGATGGCACTACTGTTTG	1210–1305	pET-32a
R2-VP60-5.1-1	GGAATTCGGTAAAGCAAGTTACCCTGG	CCCAAGCTTGAGCAATTTGGGAGATGGGGTT	898–996	pET-32a
R2-VP60-5.1-2	GGAATTCTCTGCAGCTGACAACCCCAT	CCCAAGCTTGGGGTACAAATGACATGTCAG	964–1029	pET-32a
2C1-1	GGAATTCGCAGCTGACAACCCCATCTCCCAAATTGCT	CCCAAGCTTGAGCAATTTGGGAGATGGGGTTGTCAGCTGC	967–996	pET-32a
2C1-2	GGAATTCGCTGACAACCCCATCTCCCAAATTGCT	CCCAAGCTTGAGCAATTTGGGAGATGGGGTTGTCAGC	970–996	pET-32a
2C1-3	GGAATTCGACAACCCCATCTCCCAAATTGCT	CCCAAGCTTGAGCAATTTGGGAGATGGGGTTGTC	973–996	pET-32a
2C1-4	GGAATTCAACCCCATCTCCCAAATTGCT	CCCAAGCTTGAGCAATTTGGGAGATGGGGTT	976–996	pET-32a
2C1-5	GGAATTCTCTGCAGCTGACAACCCCATCTCCCAAATT	CCCAAGCTTGAATTTGGGAGATGGGGTTGTCAGCTGCAGA	964–993	pET-32a
2C1-6	GGAATTCTCTGCAGCTGACAACCCCATCTCCCAA	CCCAAGCTTGTTGGGAGATGGGGTTGTCAGCTGCAGA	964–990	pET-32a
2C1-7	GGAATTCTCTGCAGCTGACAACCCCATCTCC	CCCAAGCTTGGGAGATGGGGTTGTCAGCTGCAGA	964–987	pET-32a
2C1-8	GGAATTCTCTGCAGCTGACAACCCCATC	CCCAAGCTTGGATGGGGTTGTCAGCTGCAGA	964–984	pET-32a
R2-VP60-4.1	GGAATTCGGTAAAGCAAGTTACCCTGGA	CCCAAGCTTGGGGAGATGGGGTTGTCAGCTGC	898–987	pET-32a
R2-VP60-4.2	GGAATTCTGGTATGCTAGTGCCGGGTC	CCCAAGCTTGGGGGTACAAATGACATGTCAGG	946–1029	pET-32a
1G2-1	GGAATTCGCCGCCATTGACCACGACAAAGGT	CCCAAGCTTGACCTTTGTCGTGGTCAATGGCGGC	877–900	pET-32a
1G2-2	GGAATTCGCCATTGACCACGACAAAGGT	CCCAAGCTTGACCTTTGTCGTGGTCAATGGC	880–900	pET-32a
1G2-3	GGAATTCATTGACCACGACAAAGGT	CCCAAGCTTGACCTTTGTCGTGGTCAAT	883–900	pET-32a
1G2-4	GGAATTCGACCACGACAAAGGT	CCCAAGCTTGACCTTTGTCGTGGTC	886–900	pET-32a
1G2-5	GGAATTCTTTGCCGCCATTGACCACGACAAA	CCCAAGCTTGTTTGTCGTGGTCAATGGCGGCAAA	874–897	pET-32a
1G2-6	GGAATTCTTTGCCGCCATTGACCACGAC	CCCAAGCTTGGTCGTGGTCAATGGCGGCAAA	874–894	pET-32a
1G2-7	GGAATTCTTTGCCGCCATTGACCAC	CCCAAGCTTGGTGGTCAATGGCGGCAAA	874–891	pET-32a

^A^Underlined letters indicate additional restriction enzyme sites: EcoRI and Hind III for the vector pET-32a, EcoRI and SalI for the vector pGEX-6P-1, and HindIII and EcoRI for the vector pcDNA3.1(+) were introduced into each primer. The boxed TTAs are the stop codon additions introduced into the antisense primers of the VP60 fragments for use with pGEX-6P-1. ^B^Nucleotide positions correspond to those in the sequence of the RHDV2 *VP60* gene (GenBank accession no.: HE800532.1).

**Table 5:** Sequences of the synthesized polypeptides.

Name	Virus strain	Sequence	Length
R-A1	RHDV1	^298^RRGSASYSGNNSTNVLQFWYAN^319^	22 aa
R2-B1	RHDV2	^298^DKGKASYPGSSSSNVLELWYAS^319^	22 aa
R-A2	RHDV1	^429^PNASAVTYTPQPDRIVTT^446^	18 aa
R2-B2	RHDV2	^429^PNSSAITYTPQPNRIVNA^446^	18 aa
R-A3	RHDV1	^405^YAVVTGTNQNPTG^417^	13 aa
R2-B3	RHDV2	^405^YGVATGINQATAG^417^	13 aa
