# Supplementary material for: Production, Characterization, and Epitope Mapping of Monoclonal Antibodies Against Different Subtypes of Rabbit Hemorrhagic Disease Virus (RHDV)
Source: Sci Rep. 2016 Feb 16;6:20857. doi: 10.1038/srep20857 (PMC4754648; doi:10.1038/srep20857)

Supplementary Information

Scientific Reports office

Production, Characterization, and Epitope Mapping of Monoclonal Antibodies Against Different Subtypes of Rabbit Hemorrhagic Disease Virus (RHDV)

Desheng Kong * , Jiasen Liu * [#], Qian Jiang, Zuo Yu, Xiaoliang Hu, Dongchun Guo， Qianqian Huang, Meihui Jiao, Liandong Qu[#]

Zoonosis of Natural Foci, State Key Laboratory of Veterinary Biotechnology, Harbin Vet Res Institute of Chinese Academy of Agricultural Sciences, Harbin 150001, PR China

＊Both authors contributed equally to this work

[#]Corresponding author: Jiasen Liu and Liandong Qu

Tel: +86 189 4606 6100; fax: +86 0451 51997176

E-mail address: neauljs@163.com

Desheng Kong < guwangdaben@163.com >

Qian Jiang < jiangqian623@sina.com.cn >

Zuo Yu < yuzuosy@163.com >

Xiaoliang Hu < liang679@163.com >

Dongchun Guo < gdongchun@126.com >

Qianqian Huang < huangqq0521@126.com >

Meihui Jiao < 15866022863@163.com >

Liandong Qu < qld@hvri.ac.cn >

Address：Harbin Vet Res Institute, Chinese Academy of Agricultural Sciences, 427 Maduan Street, Nangang District, Harbin 150001, PR China

Supplementary Figure Legends

Fig. S1 Western blot detection of recombinant His-tagged VP60 of RHDV1 (pVP60-1) and RHDV2 (pVP60-2) with rabbit

hyperimmune serum. M: PageRuler Prestained Protein Ladder (Thermo Scientific, USA).

Fig. S2 IFA detection of recombinant VP60 by eukaryotic expression. 48h after infection or transfection, green fluorescence were both detected in the Sf9 and Hela cells. Demonstration of VP60 expressing cells in IFA with rabbit hyperimmune serum in Sf9 expressed sVP60-1 (A) and sVP60-1 (B) or Hela expressed eVP60-1 (D) and eVP60-1 (E). Sf9 infected wild type baculovirus (C) and Hela transfected with pcDNA3.1(+) (F) were used as negative controls.

Fig. S1

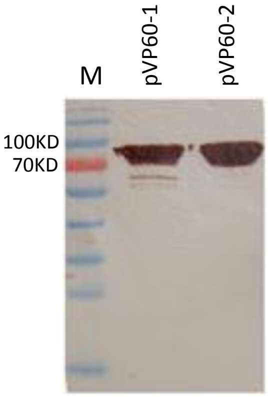

Fig. S2

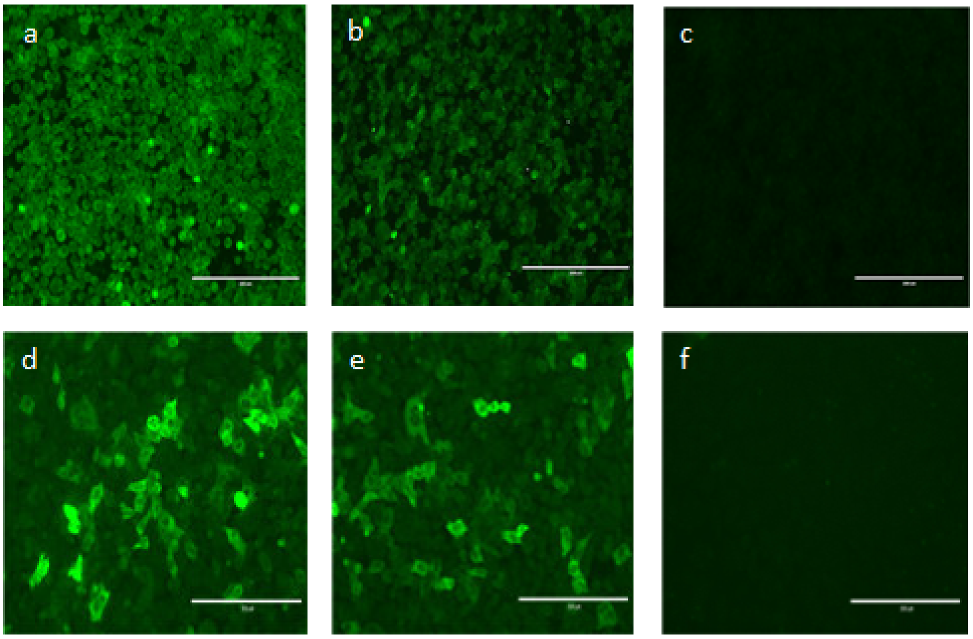

Supplement: Supplementary Information [file srep20857-s1.pdf]
